# A Framework for Composite Layup Skill Learning and Generalizing Through Teleoperation

**DOI:** 10.3389/fnbot.2022.840240

**Published:** 2022-02-11

**Authors:** Weiyong Si, Ning Wang, Qinchuan Li, Chenguang Yang

**Affiliations:** ^1^Bristol Robotics Laboratory, Faculty of Environment and Technology, University of the West of England, Bristol, United Kingdom; ^2^Faculty of Mechanical Engineering and Automation, Zhejiang Sci-Tech University, Hangzhou, China

**Keywords:** semi-autonomous composite layup, human-in-the-loop, dynamic movement primitives, learning from demonstration, teleoperation

## Abstract

In this article, an impedance control-based framework for human-robot composite layup skill transfer was developed, and the human-in-the-loop mechanism was investigated to achieve human-robot skill transfer. Although there are some works on human-robot skill transfer, it is still difficult to transfer the manipulation skill to robots through teleoperation efficiently and intuitively. In this article, we developed an impedance-based control architecture of telemanipulation in task space for the human-robot skill transfer through teleoperation. This framework not only achieves human-robot skill transfer but also provides a solution to human-robot collaboration through teleoperation. The variable impedance control system enables the compliant interaction between the robot and the environment, smooth transition between different stages. Dynamic movement primitives based learning from demonstration (LfD) is employed to model the human manipulation skills, and the learned skill can be generalized to different tasks and environments, such as the different shapes of components and different orientations of components. The performance of the proposed approach is evaluated on a 7 DoF Franka Panda through the robot-assisted composite layup on different shapes and orientations of the components.

## 1. Introduction

**Figure 1:**
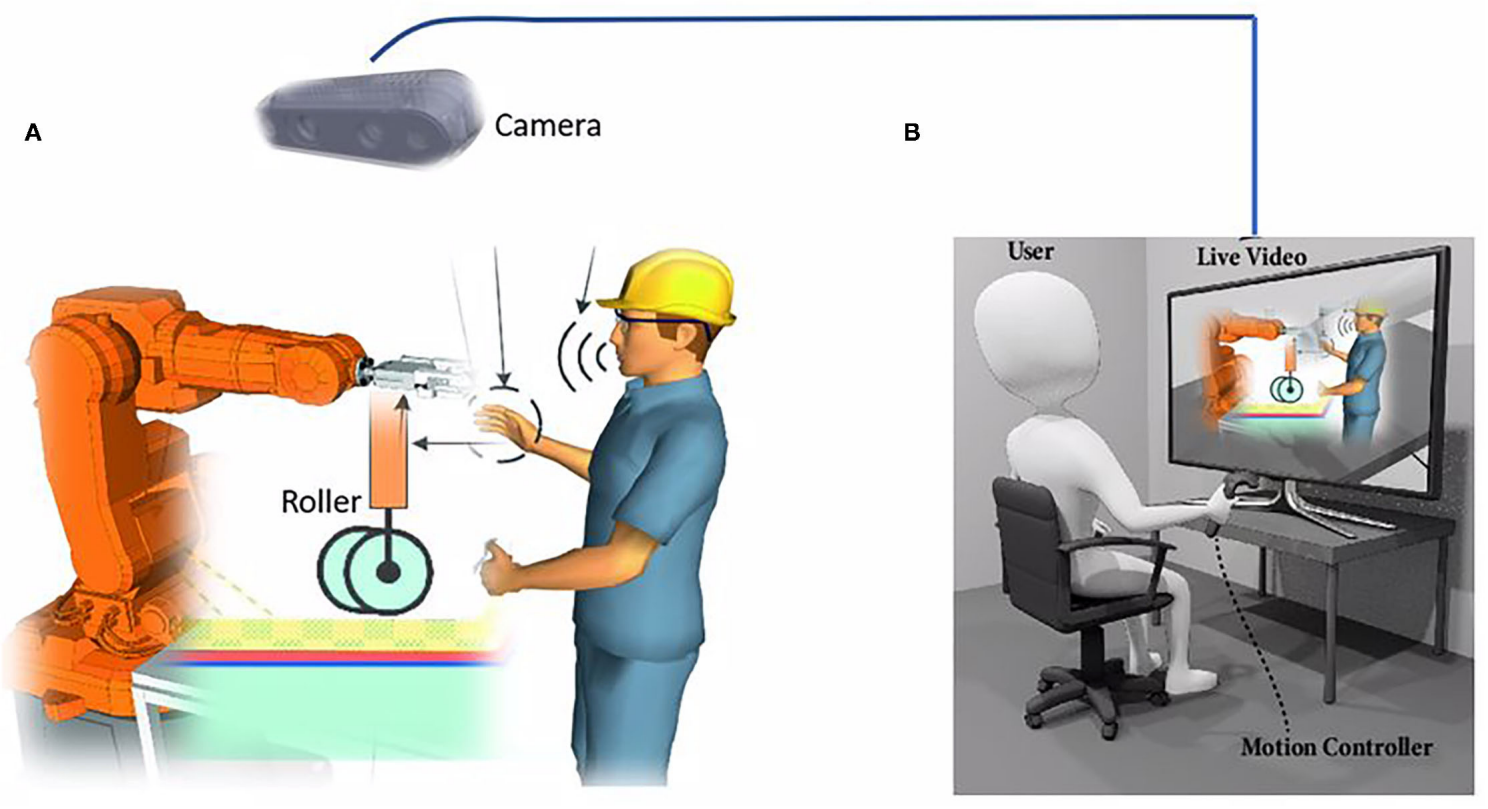
**(A)** shows a robot-assisted composite layup, and an in-site assisted person can collaborate with the robot and a demonstration expert in **(B)**. A camera in **(A)** provides visual feedback for the human operator sitting in **(B)**, who can teleoperate the robot manipulator to execute composite layup based on the visual feedback. **(B)** presents the teleoperation scenario, in which the human operator commands the robot based on the visual feedback and the teleoperated device. The two people can collaborate to perform the composite layup and transfer the composite layup skills to the robot manipulator.

Currently, robots have been widely used in various fields, such as an industrial plant (Björnsson et al., 2018; Lamon et al., 2019; Rodrıguez et al., 2019; Raessa et al., 2020), medical healthcare (Tavakoli et al., 2020; Yang et al., 2020), rehabilitation exoskeleton (Li et al., 2019, 2020), space exploration (Papadopoulos et al., 2021) and it has great advantages on the repetitive accuracy and reducing the cost. Nowadays, robots are expected to perform more challenging tasks, such as medical scanning, and composite layup as shown in Figure 1. These tasks often feature contact-rich manipulation and significant uncertainty of the different tasks, such as variance among the products in flexible manufacturing. Although our humans do not understand the principle behind manipulation, humans have the amazing capability to deal with the uncertainty and complexity in these tasks (Zeng et al., 2021). Therefore, roboticists proposed to make the robot learn the manipulation skills from humans. One of the main problems is how to learn complex and human-like manipulation skills. This study aims to develop a human-robot skill transfer system based on teleoperation and propose an approach to transfer human skills to robots. Figure 1 illustrates the composite layup process through teleoperation and human-robot collaboration. It is challenging to transfer the manipulation skill to robots through teleoperation efficiently and intuitively (Si et al., 2021c).

Collaborative robots have been increasingly important in manufacturing, such as the human-robot interaction and collaboration. Especially for the flexible manufacturing, small-batch and variance among the components put forward new requirements for traditional industrial robots in the smart factory. The main challenge is the flexibility of the manufacturing system, which allows the system to react to the changes of the new products. The human-robot skill transfer has proved a potential solution for flexible manufacturing systems (Ochoa and Cortesao, 2021; Yang et al., 2021b). To realize lightweight structures with high performance, composites have been widely used in several industries, such as aerospace, automotive, and construction etc. Carbon fiber is the main raw material of composite material production. Currently, for low-volume production and complex parts, a hand layup is still the main method, which laminates plies of carbon fiber prepreg (Malhan et al., 2021). The hand layup process is ergonomically challenging and skill-intensive. Human operators must apply various levels of pressure to the plies. In addition, sometimes several people need to collaborate to conform larger plies to complex contours. However, the hand layup process is labor-intensive and can exhibit inconsistency due to variability in human operation. Sheet layup automation can reduce ergonomic challenges, increase the production efficiency, and ensure processing quality (Malhan et al., 2020).

As machine learning techniques have been employed in various areas, such as image recognition, distributed wireless sensor networks (Lu et al., 2021b), and natural language process, the data-driven methods have proved to have several benefits, especially the generalization capability, for robot skill learning. Nowadays, robot skill learning gained much attention, and machine learning methods have been used in robot learning. For example, reinforcement learning (RL), especially the deep reinforcement learning (DRL) method, has shown powerful generalization capability for complex manipulation skills (Kroemer et al., 2021). Deep learning techniques can investigate the powerful capability for the multimodal perceptual information; hence, the DRL equipped the RL with an enhanced representation capability for multimodal information, such as vision and haptic information. However, the limitation of the DRL is the low learning efficiency and relying on big data. It is hard and expensive to acquire massive training data for robot skill learning in practice. Researchers proposed to train the manipulation skill on the simulator and then the transfer learning technique was employed to deploy the learned skill in real robots. However, the high-accuracy simulation environment for contact manipulation tasks is hard to attain, since the friction, stiffness, and damping of the contact process is hard to model. Learning from demonstration (LfD) is a feasible solution for robot skill learning and human-robot skill transfer (Wang et al., 2020).

Robot skill LfD can be categorized into two branches, based on the dynamic system method and statistical methods (Ravichandar et al., 2020). The dynamic system based methods, such as dynamic movement primitives (DMPs) (Ijspeert et al., 2013) and autonomous systems, can guarantee the converge of the skills, which is significantly important for physically human-robot interaction and critical safety tasks, such as medical scanning. In order to enhance the encoding capability of the dynamic system based model, it has been extended into non-Euclid space, such as stiffness, quaternion based orientation skill, and manipulability skills (Yang et al., 2018; Ravichandar et al., 2020). The statistical methods, such as Gaussian mixture model (GMM)/Gaussian mixture regression (GMR), hidden semi-Markov models (HSMM), probabilistic movement primitives (ProMP), could benefit the statistical property to make the best use of the multiple demonstration data and model the multiple modal information (Zeng et al., 2020). Currently, the combined methods have been proposed to make use of the advantages of the convergence guarantee of dynamic system based method and the statistical property of the statistical methods.

The main contributions of this study can be summarized as follows: 1) We developed a human-robot skill transfer system consisting of a 3D mouse device as the teleoperation interface, a 7 DoF Franka Emika robot manipulator, and a Realsense camera for visual feedback. Additionally, the composite layup task was used to evaluate the performance of the system. 2) DMPs were used to model the primitive motion skills as high-level “bricks” of the complex task. A complex task is parameterized into several motion primitives represented by the parameters of primitive motion skill; hence, combining and re-organizing the motion primitives can form a complex trajectory, which allows generalizing the learned skill to novel tasks and environments. 3) The human-robot skill transfer based on the proposed system provides a solution for robot skill learning through teleoperation or human-robot collaboration. The proposed method is more suitable for human-robot skill transfer in hazardous environments or situations that humans cannot access, such as nuclear waste disposal, lockdown under the pandemic.

The rest of this article is organized as follows. Section II presents the most related previous studies on LfD through teleoperation and motion primitive method. Section III presents basic knowledge of the dynamics of robot manipulators, null-space optimization, and the DMPs. The methodology is detailed in Section IV, followed by the experiments in Section V. Finally, Section VI concludes this article.

## 2. Related Work

### 2.1. Teleoperation for Human-Robot Skill Transfer

In terms of algorithms for LfD, there are generally two types, offline and online learning. Learning from demonstration through teleoperation could provide solutions to both types, offline learning and online learning. In Peternel et al. (2016), the authors proposed a human-in-the-loop paradigm to teleoperate and demonstrate a complex task to a robot in real-time. However, this work did not consider the compliant manipulation skills. Online LfD has some advantages over offline learning from demonstration. First, online LfD could form the skill model gradually during the demonstrations. Second, the transition between the teleoperated mode, semi-autonomous and autonomous mode is straightforward. Third, online learning also could provide real-time feedback on the performance of the model, which is like the iterative learning control. The demonstrator could get real-time feedback on the performance of the learned skills. In addition, the learned skill could execute the task online and directly on the sensorimotor level. Finally, because the skill is encoded in the end-effector, it is straightforward to transfer among different robot platforms.

In Latifee et al. (2020), incremental learning from the demonstration method was proposed based on the kinaesthetic demonstration to update the current learned skill model. But the kinaesthetic teaching method lacks immersive, especially involved contact-rich task with tactile sensing. Also, it is hard to demonstrate the impedance skill simultaneously. During the human-in-the-loop demonstration, there is a requirement on the mechanism of control allocation and adaptation between the human demonstration and the autonomous execution by the robot.

In Rigter et al. (2020), the authors integrated shared autonomy, LfD, and RL, which reduced the human effort in teleoperation and demonstration time. The controller can switch between autonomous mode and teleoperation mode, enabling controller learning online. The human-in-the-loop provides a solution for imitation learning to exploit human intervention, which can train the policy iteratively online (Mandlekar et al., 2020). Shared control is an approach enabling robots and human operators to collaboratively efficiently. In addition, shared control integrated with LfD can further increase the autonomy of the robotic system, which enables efficient task executions (Abi-Farraj et al., 2017). Human-in-the-loop and learning from the demonstration were used to transfer part assembly skills from humans to robots (Peternel et al., 2015). An approach combining operator's input and learned model online was developed for remotely operated vehicles (ROVs) to reduce human effort and teleoperation time. In addition, intelligent control methods were employed in the teleoperation to improve the trajectory tracking accuracy, which can ensure the stability of the human-robot skill transfer system (Yang et al., 2019, 2021a). A comprehensive review on human-robot skill transfer can refer to Si et al. (2021c).

### 2.2. Motion Primitives

In the past years, many researchers from the motor control and neurobiology field tried to answer how biological systems execute complex motion in versatile and creative manners. The motion primitives theory was proposed to answer this question, which means our humans can generate a smooth and complex trajectory out of multiple motion primitives. DMPs are an effective model to encode the motion primitives for robots. Therefore, how to generate a smooth and complex trajectory based on a library of DMPs, has gained attention in the robot skill learning communities, and several approaches have been developed to merge the DMPs sequences. In Saveriano et al. (2019), the authors proposed a method to merge motion primitives in Cartesian space, including position and orientation parts. The convergence of the merging strategy has proved theoretically, and experimental evaluation was performed as well.

Additionally, the motion primitives theory and knowledge-based framework were integrated and employed in the surgery, which can be generalized to different tasks and environments (Ginesi et al., 2019, 2020). Furthermore, the sequence of DMPs was employed to encode cooperative manipulation for mobile dual-arm (Zhao et al., 2018). The authors proposed to build a library of motion primitive through LfD, and the library, including the translation and orientation, can be generalized to different tasks and novel situations (Pastor et al., 2009; Manschitz et al., 2014). Also, a novel movement primitive representation, named Mixture of attractors, was proposed to encode complex object-relative movements (Manschitz et al., 2018). In our previous study, we proposed a method to merge the motion primitive based on the execution error and real-time feedback to improve the generalization capability and robustness (Si et al., 2021b).

## 3. Preliminary

### 3.1. Robot Dynamics

The general form of dynamics of the n-DOF serial manipulator robot can be modeled as Santos and Cortesão (2018),


(1) $$\begin{array}{l} D\left(q\right)\ddot{q}+C\left(q,\dot{q}\right)\dot{q}+G\left(q\right)+\tau^{ext}=\tau_{c} \end{array}$$

where the *D*(*q*) is the inertia matrix, the $C\left(q,\dot{q}\right)$, and *G*(*q*) represent the Coriolis and centrifugal, respectively. *q* and $\dot{q}$ are the joint position and velocity in the joint space, respectively. τ_*c*_ is the actuator torque and τ^*ext*^ represents torque generated by the end-effector interacting with environments.


(2) $$\begin{array}{l} \tau_{c}=\tau^{ext}+C\left(q,\dot{q}\right)\dot{q}+G\left(q\right)+\tau_{cmp} \end{array}$$

Based on Equations (1) and (2), the designed control variable can be described as the following,


(3) $$\begin{array}{l} \tau_{cmp}=D\left(q\right)\ddot{q} \end{array}$$

where τ_*cmp*_ represents a new control variable in the joint space. In order to facilitate the following analysis, we design the controller in Cartesian space; hence, Equation (3) is rewritten in Cartesian space as the follows,


(4) $$\begin{array}{l} m_{p}\left(q\right){ẍ}_{p}-m_{p}\left(q\right)\dot{J}\left(q\right)\dot{q}=f_{tol} \end{array}$$

(5) $$\begin{array}{l} m_{p}\left(q\right)={\left(J\left(q\right)D^{-1}\left(q\right)J^{T}\left(q\right)\right)}^{-1} \end{array}$$

where *m*_*p*_(*q*) represents the inertial matrix in Cartesian space, *f*_*tol*_ is the control force in Cartesian space, and *J*(*q*) is the Jacobian matrix. The task space velocity can be described as,


(6) $$\begin{array}{l} {ẋ}_{p}=J\left(q\right)\dot{q} \end{array}$$

The ẋ_*p*_ is the velocity in Cartesian space. Additionally, the control torque τ_*cmp*_ can be described as,


(7) $$\begin{array}{l} \tau_{cmp}=J^{T}\left(q\right)f_{tol} \end{array}$$

### 3.2. Null-Space Optimization

For the redundant manipulator, the null space can be used to execute second priority tasks, such as obstacle avoidance, tracking orientation, and pose optimization. The property of the null space has a lot of benefits, such as the control torque will not influence the main task. In this study, we optimize the robot pose to keep the joint close to the middle of the range of the joint. The total torque employed in the joint can be described as,


(8) $$\begin{array}{l} \tau_{cmp}=\tau_{m}+\tau_{null} \end{array}$$

where τ_*null*_ is the optimization torque in the null space, and τ_*m*_ is the torque for the main task. The τ_*null*_ can be represented as,


(9) $$\begin{array}{l} \tau_{null}=N_{pro}^{T}\left(q\right)\tau_{n} \end{array}$$

where τ_*n*_ represents the optimization torque, and $N_{pro}^{T}\left(q\right)$ is the null space projector. Because the τ_*null*_ is executed in the null-space, the optimization task will not affect the main task. The null space projector $N_{pro}^{T}\left(q\right)$ can be described as,


(10) $$\begin{array}{l} N_{pro}^{T}\left(q\right)=\left[I-J^{T}\left(q\right)J^{+}{\left(q\right)}^{T}\right] \end{array}$$

where *I* is a identity matrix, and the *J*^+^(*q*) is the inverse of *J*(*q*), which can be described as,


(11) $$\begin{array}{l} J^{+}\left(q\right)=D^{-1}\left(q\right)J^{T}\left(q\right)m_{p}\left(q\right) \end{array}$$

where *m*_*p*_(*q*) represents the inertial matrix in Cartesian space, *J*(*q*) is the Jacobian matrix. *D*(*q*) is the inertia matrix. The optimization torque can be computed as,


(12) $$\begin{array}{l} \tau_{n}=D\left(q\right)K_{o}\frac{\partial Q\left(q\right)}{\partial q} \end{array}$$

*K*_*o*_ is a gain matrix, which needs to be designed based on the requirement on the pose optimization and main tasks. *Q*(*q*) is the cost function, which tries to control the joint as close as the middle of the joint angle range.


(13) $$\begin{array}{l} Q\left(q\right)=-\frac{1}{2}\overset{n}{\underset{i=1}{\sum }}{\left(\frac{q_{i}-q_{id}}{q_{imax}-q_{imin}}\right)}^{2} \end{array}$$

where *q*_*i*_ is the current angle of the ith joint, *q*_*id*_ is the middle angle of ith joint. *q*_*imax*_ and *q*_*imin*_ are the maximum and the minimum angle of ith joint.

### 3.3. Dynamic Movement Primitives

Dynamic movement primitives is an effective model for encoding motion skills *via* a second-order dynamical system with a nonlinear forcing term. The core idea of robots skills based on DMPs is to model the forcing term in such a way, allowing to generalize the learned skills to a new start and goal position while maintaining the shape of the learned trajectory. DMPs can be used to model both periodic and discrete motion skills. Currently, most research on DMPs mainly focuses on the position and orientation DMPs and their modifications (Lu et al., 2021a; Si et al., 2021a), which can be used to represent arbitrary movements for robots in Cartesian or joint space by adding a nonlinear term to adjust the shape of trajectory. Also, the DMPs can be used to model the force profiles (Zhang et al., 2021) and stiffness profiles (Zeng et al., 2018). For one degree of multiple dimensional dynamical systems, the transformation system of position DMP can be modeled as follows (Ijspeert et al., 2013),


(14) $$\begin{array}{l} \tau_{s}\dot{v}=\alpha_{z}\left(\beta_{z}\left(p_{g}-p\right)-v\right)+F_{p}\left(x\right) \end{array}$$

(15) $$\begin{array}{l} \tau_{s}ṗ=v \end{array}$$

where the *p*_*g*_ is the desired position, *p* is the current position; *v* is the scaled velocity, τ_*s*_ is the temporal scaling parameter, which can be used to modify the velocity. α_*z*_, β_*z*_ are the design parameters, generally, α_*z*_ = 4β_*z*_. *F*_*p*_(*x*) is the nonlinear forcing term responsible for tuning the shape of the trajectory. The *F*_*p*_(*x*) can be approximated by a set of radial basic functions,


(16) $$\begin{array}{l} F_{p}\left(x\right)=\frac{\sum_{i=1}^{N}\psi_{i}\left(x\right)w_{i}}{\sum_{i=1}^{N}\psi_{i}\left(x\right)}x\left(p_{g}-p_{0}\right) \end{array}$$

(17) $$\begin{array}{l} \psi_{i}\left(x\right)=\exp\left(-h_{i}{\left(x-c_{i}\right)}^{2}\right) \end{array}$$

where ψ_*i*_(*x*) is a Gaussian radial basis function with the center *c*_*i*_ and width *h*_*i*_; *p*_0_ is the initial position, *w*_*i*_ is the weight LfD. The phase variable *x* is determined by the canonical system, which can be represented as follows,


(18) $$\begin{array}{l} \tau_{s}ẋ=-\alpha_{x}x,\;x\in \left[0,1\right];\;x\left(0\right)=1 \end{array}$$

where α_*x*_ is a positive gain coefficient, τ_*s*_ is the temporal scaling parameter, and the *x*_0_ = 1 is the initial value of *x*, which can converge to zero exponentially. For the multiple Degree of Freedom (DoF) dynamic system, each dimension can be modeled by a transformation system, but they share a common canonical system to synchronize them.

## 4. Methodology

### 4.1. Task-Space Formulation

**Figure 2:**
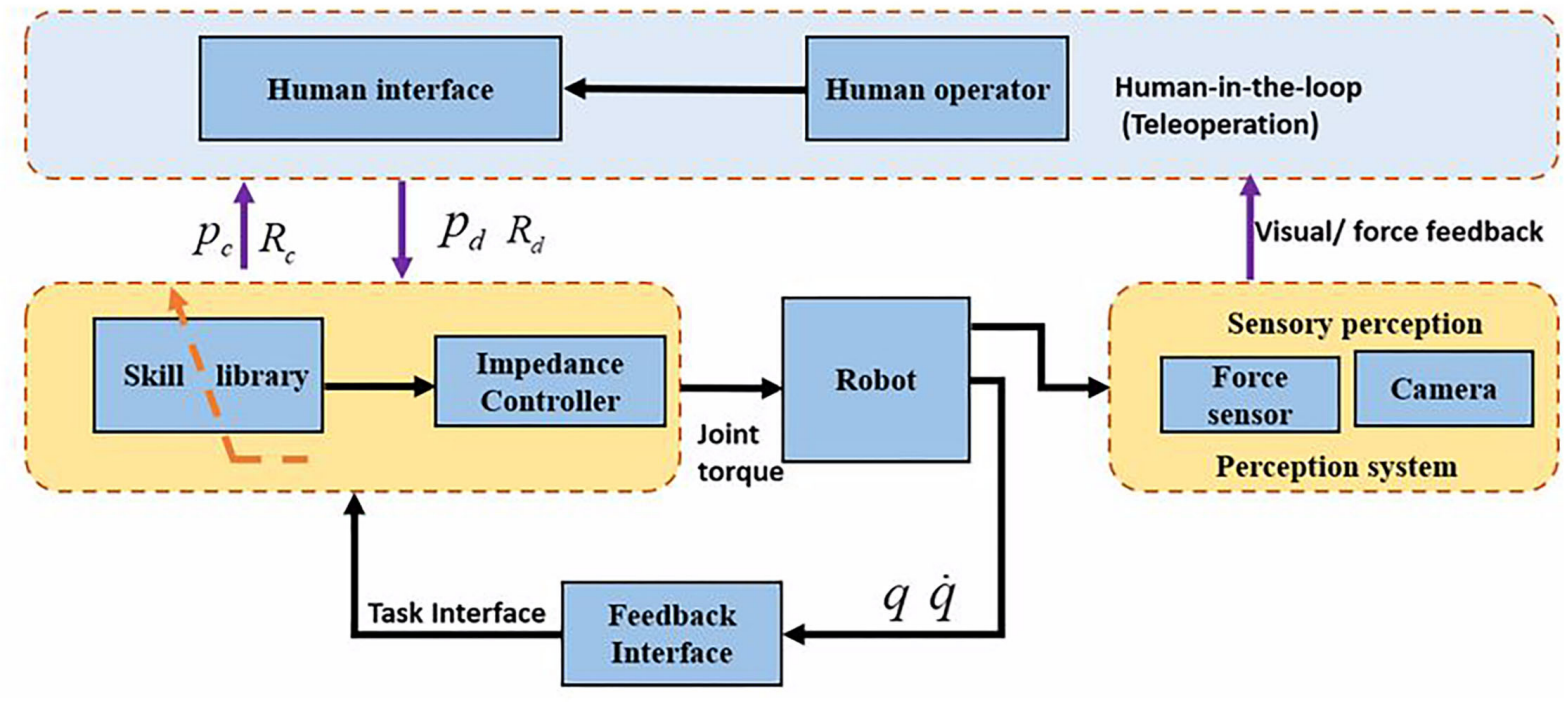
The diagram of the proposed framework for human-robot skill transfer through the human-in-the-loop. The human-in-the-loop module is the teleoperation based subsystem, which could command the robot through the teleoperation interface, a 3D mouse, and receive visual and force feedback from the perception subsystem. The impedance controller can generate joint torque command for the robot either through the teleoperation or autonomous mode (through skill library).

**Figure 3:**
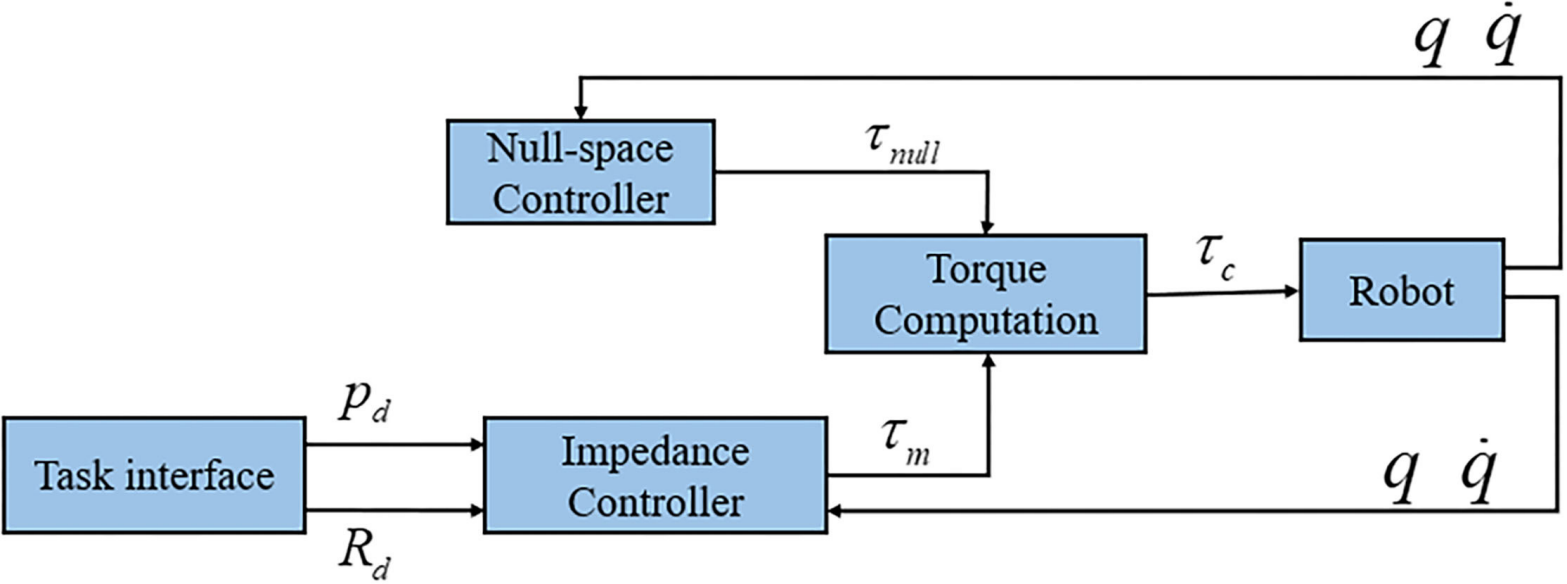
The diagram of the impedance-based control system. The task interface module generates the desired position and orientation through teleoperation or learned skill model. The impedance controller is used to track the desired position and orientation. The null-space controller is used to optimize the joint pose by using redundancy to keep the joint angle close to the middle value.

As shown in Figure 2, the proposed framework includes teleoperation, perception, skill modeling and robot control. This section derives the controller in Cartesian space, and the whole control structure can be found in Figure 3. It will be convenient to design the controller in the task space because the teleoperation control would be intuitive and straightforward in the task space. For the human-robot collaboration task, the safety of the human is significant; hence, compliant control has been employed for the collaborative robots. In 1985, impedance control was first proposed by Hogan (1985), and since then, a lot of exciting work has been done. A number of work on the impedance control was done for the manipulator control. Additionally, in order to achieve human-like manipulation, the variable impedance control was employed in the human-robot interaction. The core idea of impedance control models the dynamic behavior of robots under disturbance from the environment. In Ochoa and Cortesao (2021), the authors proposed a similar impedance controller for the polishing task. The impedance controller can be described as the following,


(19) $$\begin{array}{l} f_{imp}=A{ẍ}_{p}+D\left({ẋ}_{p}-{ẋ}_{d}\right)+K\left(x_{p}-x_{d}\right) \end{array}$$

where *A* is the mass matrix, *D* is the damping matrix, and *K* is the stiffness matrix. *x*_*d*_ represents the equilibrium point and *x*_*p*_ is the current position of the robot end-effector. *f*_*imp*_ is the interaction force between the robot end-effector and the environment. For the dynamic equation of manipulator in the task space, Equation (4), the total force *f*_*tol*_, exerting on the robot, can be calculated as follows:


(20) $$\begin{array}{l} f_{tol}=f_{c}+f_{imp} \end{array}$$

We define a new control variable $f_{tol}^{*}$, and the *f*_*c*_ is represented as follows,


(21) $$\begin{array}{l} f_{c}=-m_{p}\left(q\right)\dot{J}\left(q\right)\dot{q}+f_{tol}^{*} \end{array}$$

The Equation (4) can be represented as follows,


(22) $$\begin{array}{l} m_{p}\left(q\right){ẍ}_{p}=f_{tol}^{*}+f_{imp} \end{array}$$

Therefore, the control law of $f_{tol}^{*}$ is,


(23) $$\begin{array}{l} f_{tol}^{*}=m_{p}\left(q\right){ẍ}_{p}-\left[A{ẍ}_{p}+D\left({ẋ}_{p}-{ẋ}_{d}\right)+K\left(x_{p}-x_{d}\right)\right] \end{array}$$

In this article, the mass matrix is approximated by Ochoa and Cortesao (2021),


(24) $$\begin{array}{l} A=m_{p}\left(q\right)={\left(J\left(q\right)D^{-1}\left(q\right)J^{T}\left(q\right)\right)}^{-1} \end{array}$$

So, the control law becomes,


(25) $$\begin{array}{l} f_{tol}^{*}=D\left({ẋ}_{d}-{ẋ}_{p}\right)+K\left(x_{d}-x_{p}\right) \end{array}$$

The joint torque for the main task can be given by,


(26) $$\begin{array}{l} \tau_{m}=J^{T}\left(q\right)f_{tol}=J^{T}\left(-m_{p}\left(q\right)\dot{J}\left(q\right)\dot{q}+f_{tol}^{*}\right) \end{array}$$

Because the $\dot{J}\left(q\right)$ has a small influence on the system, the $-m_{p}\left(q\right)\dot{J}\left(q\right)\dot{q}$ can be ignored. Therefore, the control law for the main task in the Cartesian space can be written as follows,


(27) $$\begin{array}{l} \tau_{m}=J^{T}f_{tol}^{*} \end{array}$$

where $f_{tol}^{*}$ can be rewritten as,


(28) $$\begin{array}{l} f_{tol}^{*}=\left[\begin{array}{c} f\\ u \end{array}\right] \end{array}$$

where *f* is the force vector for translation control in Cartesian space, and *u* is the torque vector for orientation control. The translation controller in discrete form can be described as the following,


(29) $$\begin{array}{l} f=-D_{p}{ṗ}_{c}+K_{p}\left(p_{d}\left[t\right]-p_{c}\left[t\right]\right)+I_{p}\left(i_{p}\left[t-1\right]+\left(p_{d}\left[t\right]-p_{c}\left[t\right]\right)\right) \end{array}$$

where *D*_*p*_ is the damping matrix, *K*_*p*_ is the stiffness matrix, and *I*_*p*_ is the integral matrix. *i*_*p*_[*t* #x02212; 1] is the integral error in the position at time [*t*−1]. *p*_*d*_[*t*] and *p*_*c*_[*t*] are the desired position and current position at time t, respectively. Similarly, the orientation controller in discrete form can be represented as,


(30) $$\begin{array}{l} u=-D_{o}w_{c}+K_{o}\Delta o_{cd}+I_{o}i_{o} \end{array}$$

where *D*_*o*_ is the damping matrix, *K*_*o*_ is the stiffness matrix, and *I*_*o*_ is the integral matrix. *w*_*c*_ is the angular velocity, Δ*o*_*cd*_ is the orientation error, and *i*_*o*_ is the integral error in orientation. Finally, based on Equations (2), (8), and (27), the total torque command can be described as the following,


(31) $$\begin{array}{l} \tau_{c}=J^{T}f_{tol}^{*}+\tau^{ext}+N_{pro}^{T}\left(q\right)\tau_{null}+C\left(q,\dot{q}\right)\dot{q}+G\left(q\right) \end{array}$$

### 4.2. Task Interface Design

The desired trajectory, including the translation and orientation, is generated from the input device based on displacement commands in the teleoperation mode. The human operator is provided with a GUI interface and a 3D mouse to monitor and control the system. The 3D mouse has two buttons and a six-DoF motion sensor. The two buttons are used to switch control modes, such as teleoperation, autonomous, and collaboration. The six-DoF motion axis of the 3D mouse is employed to generate the reference trajectory for the impedance controller in Cartesian space.


(32) $$\begin{array}{l} \Delta Z={\left[\begin{array}{cc} \Delta P & \Delta R \end{array}\right]}^{T} \end{array}$$

where Δ*P* and Δ*R* represent the translational and rotational displacements, respectively. Δ*Z* is then converted to the desired motion in the robot's base frame.

In the autonomous mode, the desired trajectory in the robot's base frame, including the translation and orientation, is generated based on the learned DMPs.


(33) $$\begin{array}{l} Z_{d}={\left[\begin{array}{cc} P_{d} & R_{d} \end{array}\right]}^{T} \end{array}$$

where *P*_*d*_ and *R*_*d*_ represent the desired trajectory in translational and rotational directions, respectively. *Z*_*d*_ is then converted to the desired motion in the robot's base frame.

## 5. Experiment Study Case

This section aims at evaluating the proposed solution, human-robot skill transfer through teleoperation, by performing composite layup for different components.

### 5.1. Experiment Setup

**Figure 4:**
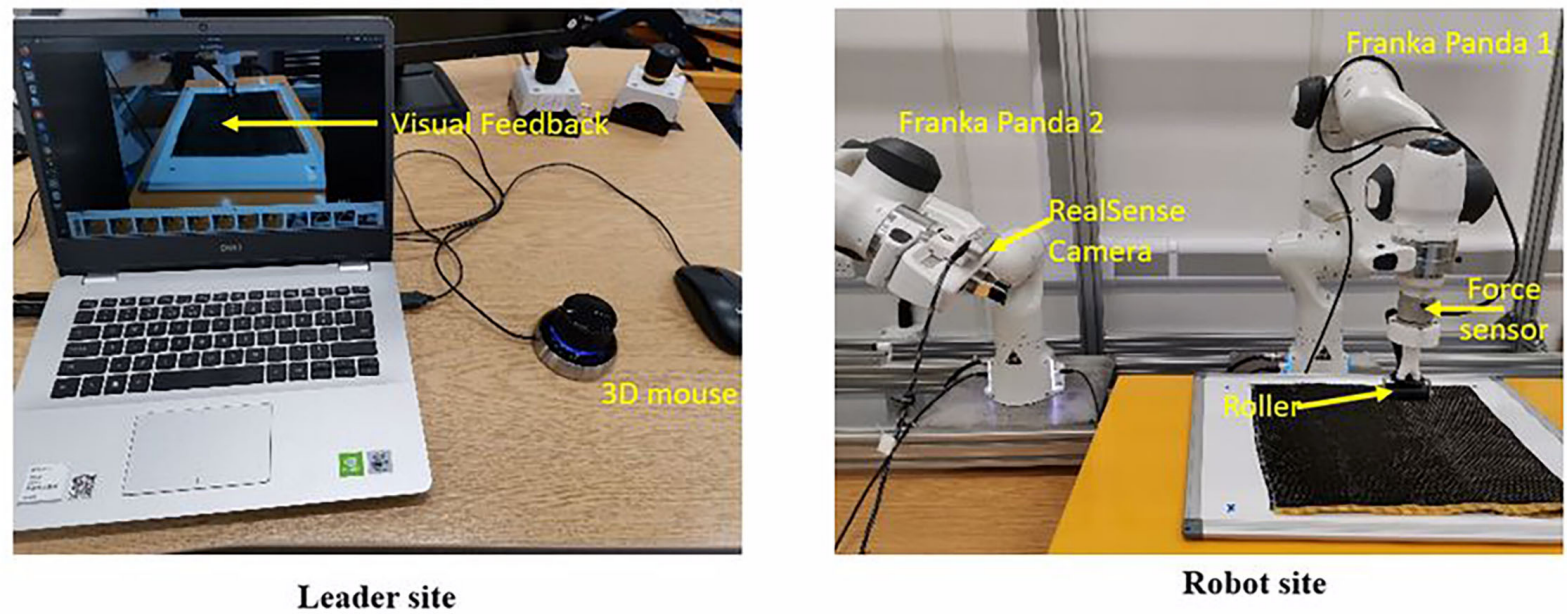
The experiment setup for the composite layup. In the leader site, the human operators teleoperate the robot to perform the composite layup.

A collaborative robot, Franka Emika Panda, was used to conduct experiments, as shown in Figure 4. An external force/torque sensor is equipped in the wrist to sense the interaction force and torque between the end-effector tool and the environment. As shown in Figure 4, we designed the fixtures (3D printing) to connect the layup tool (roller), force/torque sensor, and the robot end-effector. A RealSense depth camera D435 is used to observe the working scenario of the robot, and the visual feedback is transmitted to the computer on the leader side for the human operator to monitor the remote scenario.

A 3D mouse from the 3DConnexion company is more suitable for teleoperation, and it can output linear and angular components of the joystick's position and the status of the two buttons. The twist command of the 3D mouse, consisting of the linear and angular components, is used to map the translation and orientation of the end-effector. The buttons states as an event-trigger signal were employed to switch control modes. There is a control interface of the robot manipulator provided by the Franka control interface (FCI) on the robot side, which provides the control interface and a fast and direct low-level bidirectional connection to the robot arm. In the leader site, there is a laptop to execute the control and learning algorithm. The generated command is transferred to the Franka control board. Linux Operating system is run on the laptop, and Robot Operating System (ROS) is used to communicate among the different modules.

### 5.2. The User Interface of the Control System

**Figure 5:**
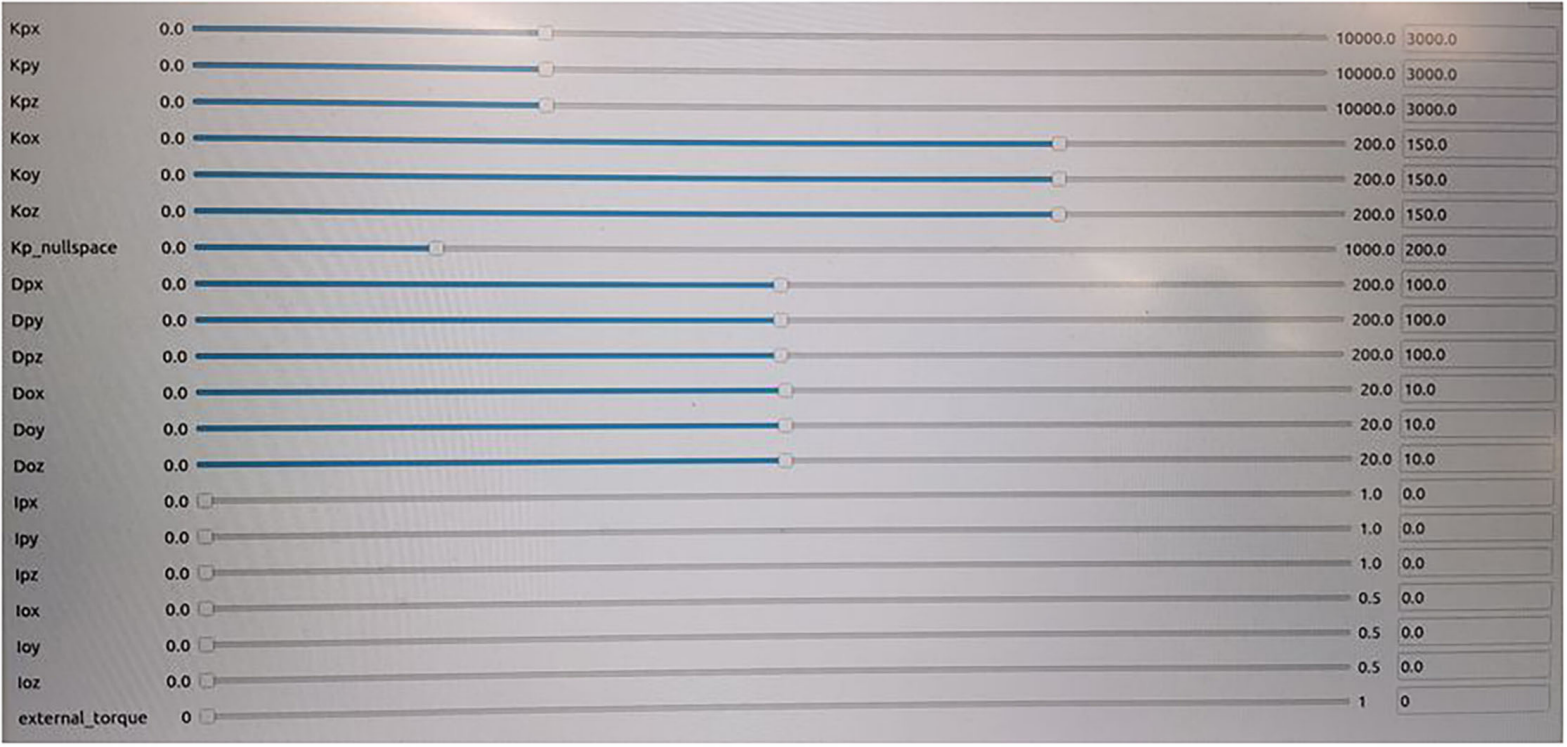
The GUI for the control system. The human operator could modify the parameters of the controller online to change the compliant behavior.

As shown in Figure 5, the control system parameters can be displayed and modified by the human operators online. The human operators can change the control modes (switching between teleoperation mode and collaboration mode) *via* adjusting these parameters, including stiffness *K*_*p*_ and *K*_*o*_, damping *D*_*p*_ and *D*_*o*_, integral *I*_*p*_ and *I*_*o*_, and the null-space optimization gain matrix *K*_*n*_. These parameters can be modified online based on the different task requirements, such as more stiff in one degree or more compliant in another degree. The interaction force/torque between the end-effector and the environment can also be displayed on a monitor, which provides more knowledge on the interaction process.

**Figure 6:**
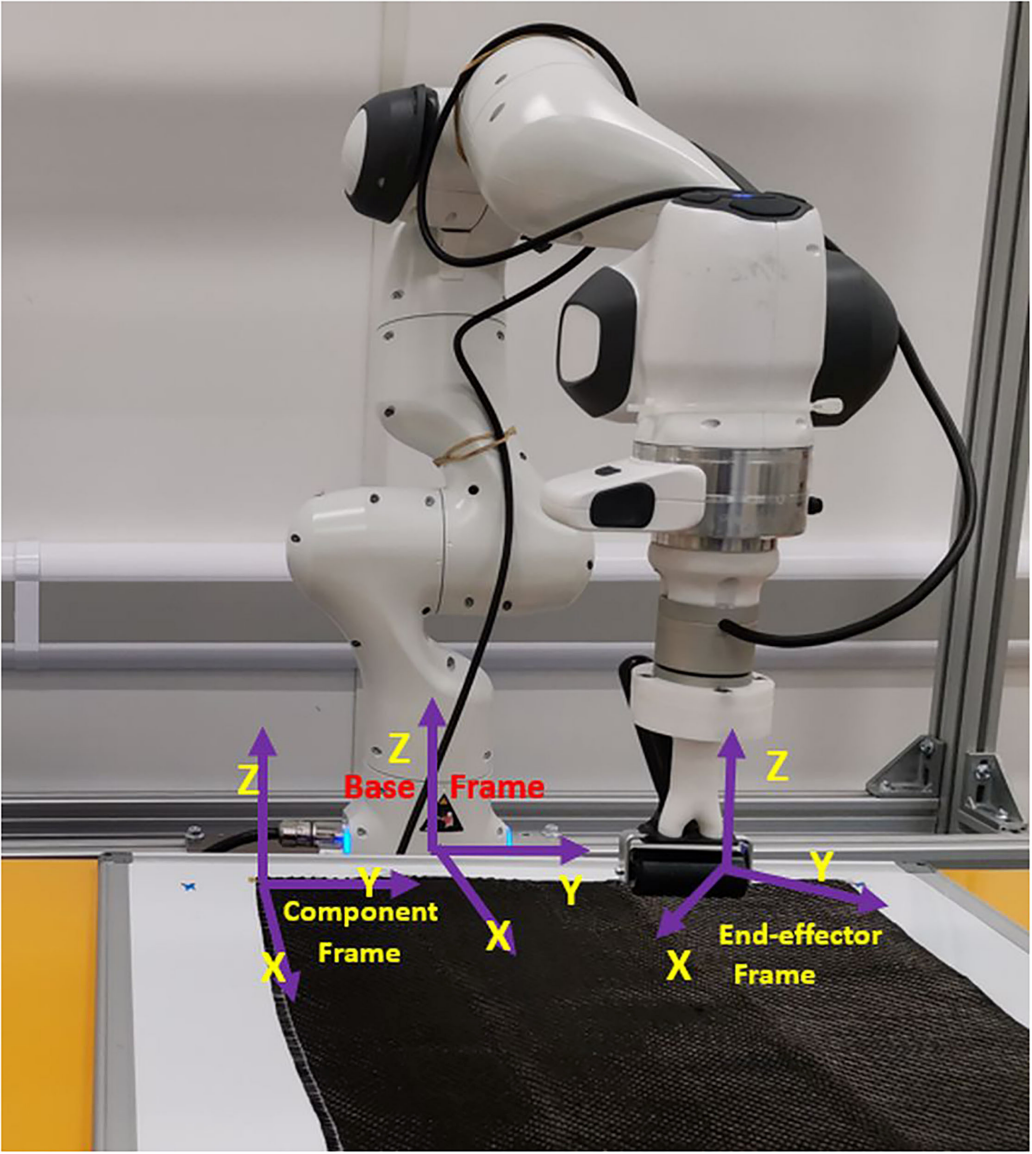
The three coordinate frames, the base frame of the robot, the end-effector frame, and the component frame.

In terms of the controller design and the human-robot skill transfer, defining the proper coordinate frame is necessary. The proper frame could reduce the cognitive workload during teleoperation and human-in-the-loop interaction and collaboration. In this study, we defined three frames on the robot sides, as shown in Figure 6. The impedance controller is defined in the cartesian space, and the desired command is based on the based frame of the robot. The task description is based on the component frame, and the transformation from the based frame of the robot to the component frame is fixed. The impedance gain defined by the user is based on the end-effector frame, therefore, the parameters of the controller need to be transformed into the based frame.

### 5.3. Human Demonstration Through Teleoperation

**Figure 7:**
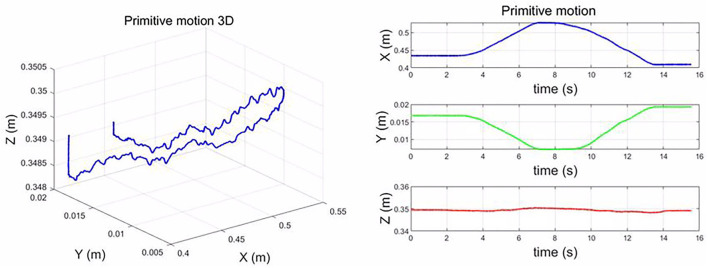
Modeling the motion primitive by dynamic movement primitive (DMP).

To enable the human-robot composite layup skill transfer, the human operator needs to composite layup through teleoperation. The motion primitives of composite layup were recorded, as shown in Figure 7. During the demonstration, the human operator demonstrated the layup for a flat component. From the results, the roller moves forward and back in the X-Y plane, which is the primitive motion skill for the composite layup. We modeled this motion primitive by DMPs, which can be generalized to different locations. Regarding the parameters of DMPs, please refer to Table 1. For the Z-axis, there is a small motion, which can be used to generate the contact force along Z-axis in the end-effector frame. The stiffness parameters for the X and Y are the same, K = 3,000, while for the Z-axis is large, K = 6,000, which can be guaranteed to generate contact force along Z-axis. The small impedance along X and Y, which can feature a compliant manner.

**Table 1:** Parameters of Dynamic movement primitives (DMPs) and the controller.

**Parameter**	**Descriptions**	**Value**
*N*	Number of Gaussian functions	100
α_*z*_	Coefficient of DMP	80
β_*z*_	Coefficient of DMP	20
τ_*s*_	Coefficient of DMP	1
*K*_*px*_,*K*_*py*_	Stiffness of position controller along X and Y axis	3,000
*K*_*pz*_	Stiffness of position controller along Z axis	6,000
*D*_*px*_,*D*_*py*_	Damping of position controller along X and Y axis	100
*D*_*pz*_	Damping of position controller along Z axis	150

### 5.4. Generalize to a Novel and Big Plane

In this case study, we would like to evaluate the generalization to a novel and large component, which is necessary for the composite layup in the real industry plant. The automation of composite layup only relies on the motion primitive and a little bit of information on the component. Generally, it is straightforward to attain the geometry of the component based on the CAD model of the part. In this case, we assumed that the vertex coordinates of the part are known. For example, given the four vertex coordinates, we can get the region where the parts need to be manufactured for a plane. For the area that needs to be processed, we divided several sub-areas. Each sub-area can be modeled with a task variable, including the start and end coordinates. For the DMPs based skill model, only the task variable is needed to reproduce the motion skills.

**Figure 8:**
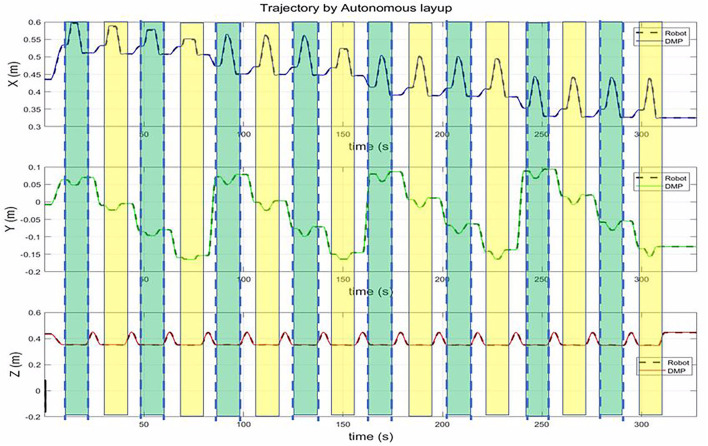
The trajectory of the roller in the autonomous mode. The green bar and the yellow bar represent the motion primitive. In this works, the motion primitive is the same, but the start and end of each motion primitives are different.

The number of motion primitive is dependent on the size of the component. For the X direction, as the generalization of DMP, the number can be random. In the Y direction, the number is the length of the workpiece, divided by the width of the roller, which ensures the roller can cover the whole workpiece. As shown in Figure 8, there are 16 motion primitives for the big plane. Each motion primitive is similar to the human demonstration motion. Between two motion primitives, we used a motion planning algorithm to generate a transition trajectory. The first row, along X-axis, shows that the coordinates of the roller decrease from 0.6 to 0.34 m. The middle row, along the Y-axis, shows that the coordinates of the roller change between −0.17 m and 0.1 m. The real trajectory of robots can cover the whole workpiece.

**Figure 9:**
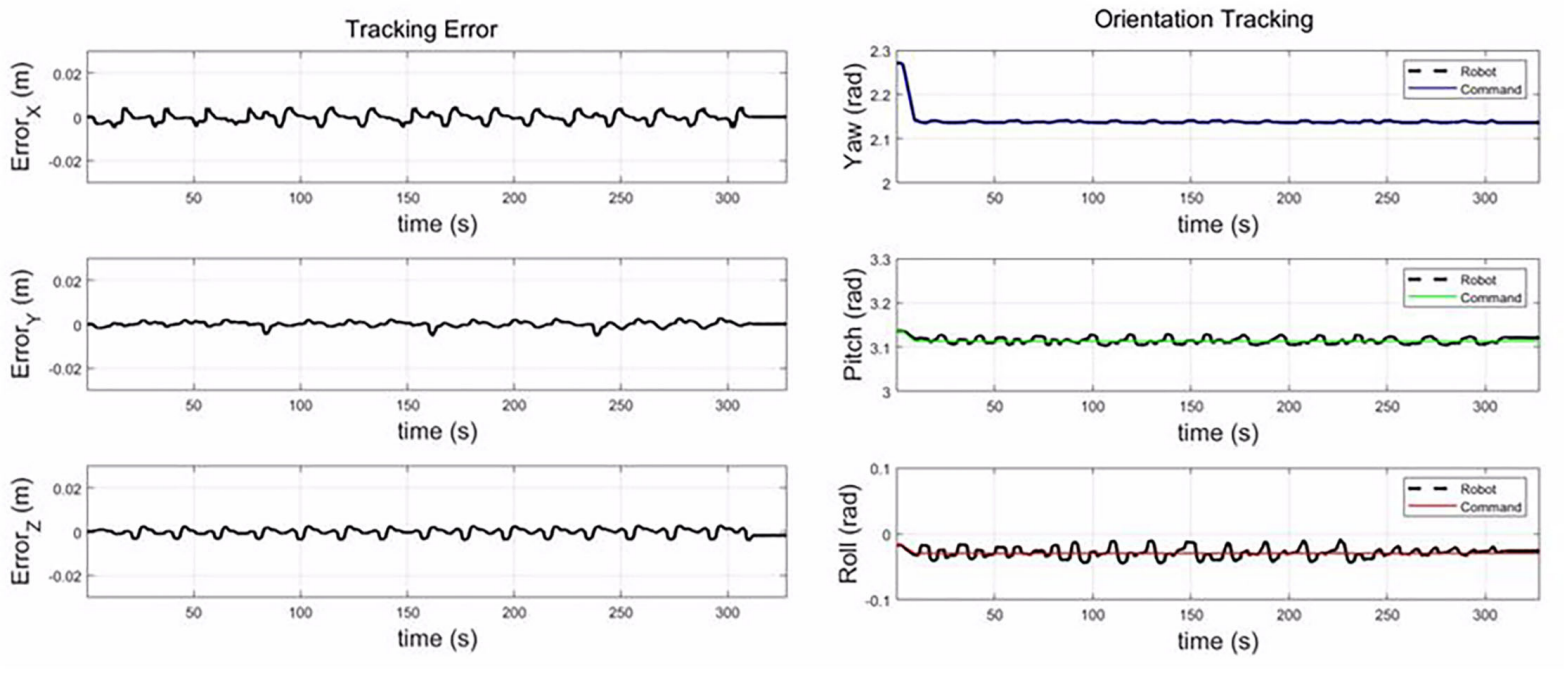
The tracking error along the X, Y, and Z-axis; and the orientation tracking in yaw, pitch, and roll.

The Figure 9 shows the tracking error between the command from the DMP model and the actual trajectory of the roller. The tracking error is less than 0.005 m in the X, Y, and Z-axis. During the composite layup, the orientation of the end-effector is fixed, and the tracking error is less than 0.02 rad. The control accuracy is enough for the composite layup, which proves the performance of the impedance controller for the composite layup.

### 5.5. Generalize to a Novel and Small Plane

In this case study, we would like to evaluate the generalization to a novel and small component, which is similar to the previous experiment case. The main difference is that the four vertex coordinates are changed. The motion primitive is the same as the previous experiment, and we evaluate that the learned skill can be generalized to a novel component with different sizes.

**Figure 10:**
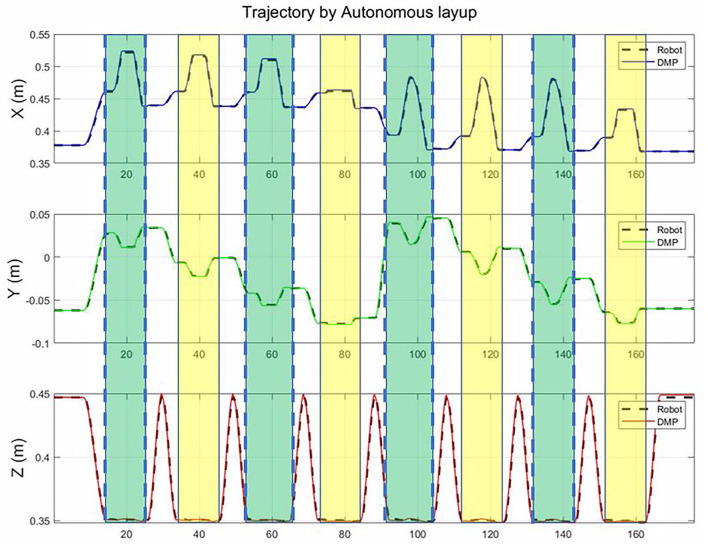
The autonomous trajectory of end-effector in the base frame of robot for novel and small plane.

**Figure 11:**
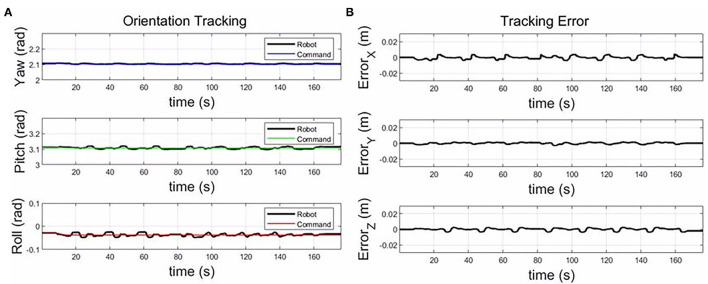
The tracking error along the X, Y, and Z-axis **(B)**; and the orientation tracking in yaw, pitch, and roll for the small plane **(A)**.

As shown in Figure 10, for the small component, only eight motion primitives are needed to cover the whole plane. The proposed framework could generalize to different sizes only based on the vertex coordinates for a plane part. From Figure 11, the tracking errors are less than 0.005 m, and the orientation tracking errors are less than 0.02 rad. The control accuracy is enough for the composite layup.

### 5.6. Generalize to a Slope Plane

This experiment case aim at evaluating the generalization to an inclined plane. In this case, the key points for this task are *P*_1_(0.36, - 0.14, 0.38), *P*_2_(0.37, 0.01, 0.42), *P*_3_(0.50, 0, 0.42), and *P*_4_(0.50, - 0.18, 0.38). We assume that we know the CAD model of the component or the orientation of the model can be measured based on sensing technology, such as machine vision.

**Figure 12:**
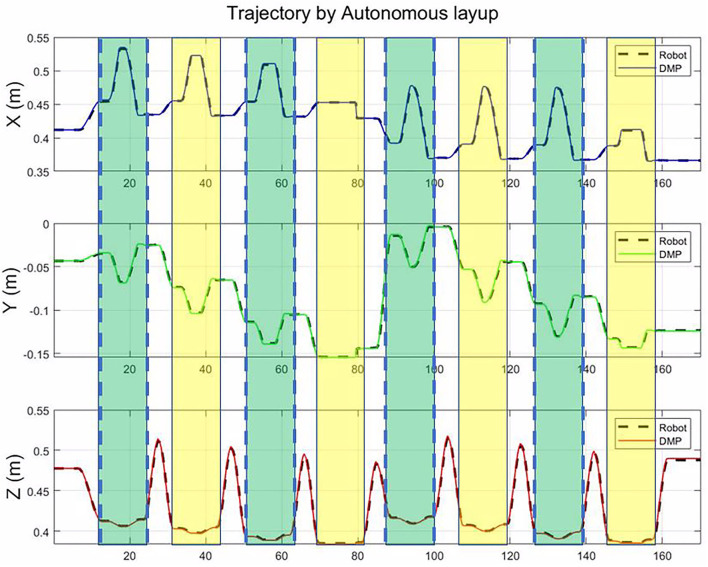
The trajectory of end-effector in the base frame of robot in autonomous mode for the slope plane.

**Figure 13:**
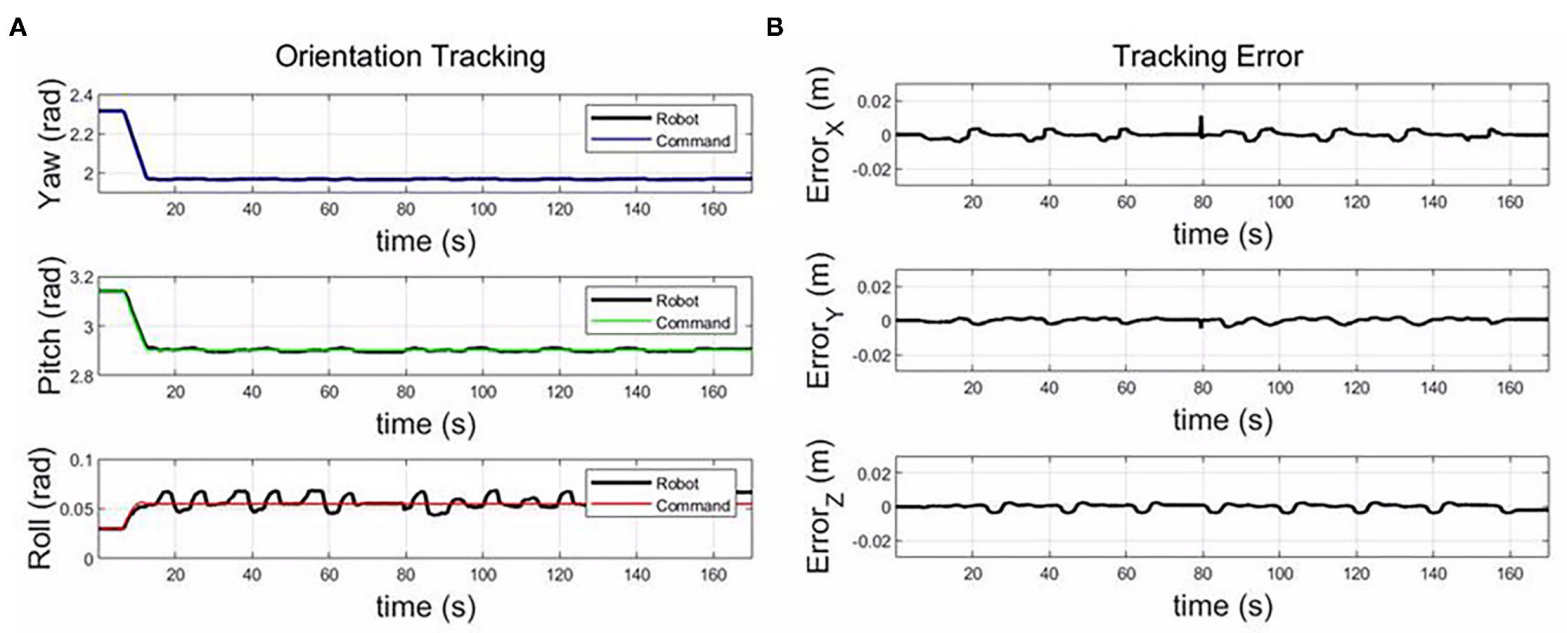
The tracking error along the X, Y, and Z-axis **(B)**; and the orientation tracking in yaw, pitch, and roll for the slope plane **(A)**.

From Figure 12, there are eight motion primitives to cover the whole inclined plane. The main differences between the inclined plane and the horizontal plane are the motion along the Z-axis and the orientation. From the third row, the motion range along the Z-axis is from 0.41 to 0.38 m in Figure 13. Additionally, the orientation is different, which needs to keep the roller perpendicular to the plane. The tracking errors are less than 0.005 m, and the orientation tracking errors are less than 0.02 rad, which demonstrates the generalization of the learned skill and the performance of the impedance-based controller for a composite layup in the inclined plane.

### 5.7. Collaboration Through Teleoperation

This experiment aims at evaluating the collaboration performance of the impedance control-based teleoperation system. Existing research focuses mostly on physical human-robot collaboration, with less work on collaboration between teleoperation, in-site humans and robots. In this experiment, we evaluated that teleoperation and in-site human can collaborate smoothly through modifying the parameters of the impedance controller. We make use of the character of the torque-computed control based on impedance control. For example, we set the stiffness of the impedance controller to a specific degree; the control along this degree becomes a free-motion mode, which can be kinesthetic teaching or adjusting the robots by an in-site human.

**Figure 14:**
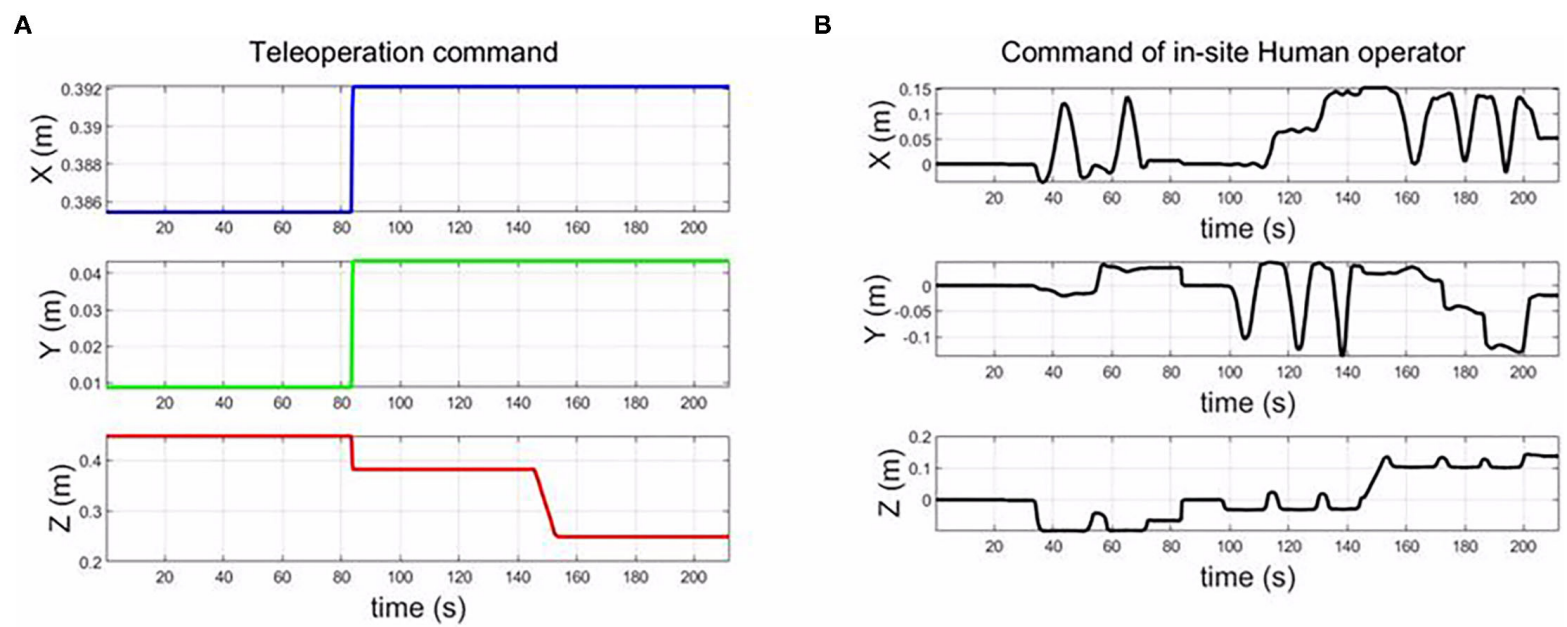
The trajectory of end-effector in the collaboration mode through teleoperation. **(A)** is the teleoperation command, and **(B)** is the input command from the in-site human operator.

**Figure 15:**
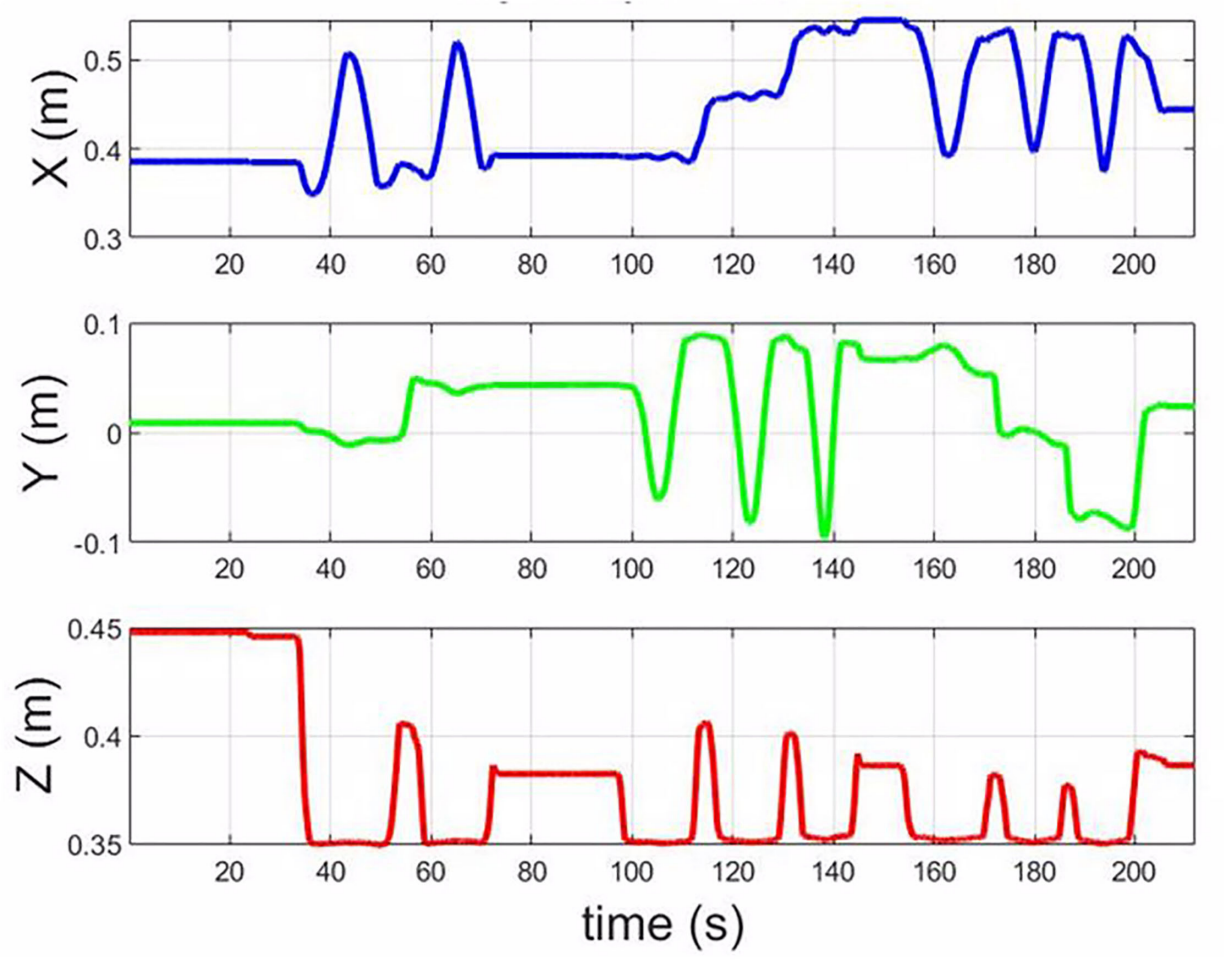
The trajectory of the end-effector in the base frame of the robot in the collaboration mode.

From Figure 14A is the control command from the teleoperation, and (Figure 14B) is the command by in-site human operation. The orientation control is autonomous by the robot. Figure 15 is the trajectory of the end-effector in the hybrid control mode. The results show that the control system can integrate teleoperation and kinesthetic demonstration and autonomous. The transition among the three modes can be smooth.

## 6. Discussion and Conclusion

In this article, a torque-computed framework based on impedance control was proposed to enable the human-robot skill transfer through teleoperation. The human user interface was developed to display the parameters of the controller and the contact force, and the human operator could modify the parameters of the control system. The 3D mouse has been used as the input device for teleoperation. In addition, because the teleoperation control design is in Cartesian space, the teleoperation mapping between the input and the robot motion is intuitive. Also, the proposed teleoperation system is compatible with other input devices, such as Omni joystick and Omge.

For the robot-assisted composite layup, the layup skills are modeled by DMPs and transferred to the robot through teleoperation. The generalization of the proposed framework has been demonstrated through different components with various sizes and orientations. Additionally, the tracking error of the impedance-based controller is less than 0.005 m, which is feasible for the composite layup. In addition, we also evaluated the performance through an experiment, teleoperation, and in-site human operator co-work. From the results, the transition is smooth. In the future, we will conduct a user study to improve the human teleoperation interface design, such as combining multiple input devices and visualizing the interaction states. We will also study the deep learning techniques to automate the perception information to investigate reactive and automated monitoring.

## Data Availability Statement

The original contributions presented in the study are included in the article/supplementary material, further inquiries can be directed to the corresponding author.

## Author Contributions

WS developed the structure of the manuscript conducted the experiments, and discussions of the results. NW mainly contributed the Preliminary and part of Introduction. CY guided the whole process of this study and writing. QL and CY critically revised the manuscript. All the authors contributed to the article and approved the submitted version.

## Funding

This study was supported in part by the Engineering and Physical Sciences Research Council (EPSRC) under grant EP/S001913 and in part by the Key Research and Development Project of Zhejiang Province under grant 2021C04017.

## Conflict of Interest

The authors declare that the research was conducted in the absence of any commercial or financial relationships that could be construed as a potential conflict of interest.

## Publisher's Note

All claims expressed in this article are solely those of the authors and do not necessarily represent those of their affiliated organizations, or those of the publisher, the editors and the reviewers. Any product that may be evaluated in this article, or claim that may be made by its manufacturer, is not guaranteed or endorsed by the publisher.

## References

[B1] Abi-Farraj F. Osa T. Peters N. P. J. Neumann G. Giordano P. R. (2017). A learning-based shared control architecture for interactive task execution, in 2017 IEEE International Conference on Robotics and Automation (ICRA) (Singapore: IEEE), 329–335.

[B2] Björnsson A. Jonsson M. Johansen K. (2018). Automated material handling in composite manufacturing using pick-and-place systems-a review. Robot Comput. Integr. Manuf. 51:222–229. 10.1016/j.rcim.2017.12.003

[B3] Ginesi M. Meli D. Nakawala H. Roberti A. Fiorini P. (2019). A knowledge-based framework for task automation in surgery. In 2019 19th International Conference on Advanced Robotics (ICAR), pages 37-42. IEEE. 10.1109/ICAR46387.2019.8981619

[B4] Ginesi M. Meli D. Roberti A. Sansonetto N. Fiorini P. (2020). Autonomous task planning and situation awareness in robotic surgery, in 2020 IEEE/RSJ International Conference on Intelligent Robots and Systems (IROS) (Las Vegas, NV: IEEE), 3144–3150.

[B5] Hogan N. (1985). Impedance control: an approach to manipulation: Part ii–implementation. J. Dyn. Sys. Meas. Control. 107, 8–16. 10.1115/1.3140713

[B6] Ijspeert A. J. Nakanishi J. Hoffmann H. Pastor P. Schaal S. (2013). Dynamical movement primitives: learning attractor models for motor behaviors. Neural Comput. 25, 328–373. 10.1162/NECO_a_00393 23148415

[B7] Kroemer O. Niekum S. Konidaris G. (2021). A review of robot learning for manipulation: challenges, representations, and algorithms. J. Mach. Learn. Res. 22, 30–31. Available online at: http://jmlr.org/papers/v22/19-804.html

[B8] Lamon E. De Franco A. Peternel L. Ajoudani A. (2019). A capability-aware role allocation approach to industrial assembly tasks. IEEE Robot. Autom. Lett. 4, 3378–3385. 10.1109/LRA.2019.2926963

[B9] Latifee H. Pervez A. Ryu J.-H. Lee D. (2020). Mini-batched online incremental learning through supervisory teleoperation with kinesthetic coupling, in 2020 IEEE International Conference on Robotics and Automation (ICRA) (Paris: IEEE), 5453–5459.

[B10] Li Z. Deng C. Zhao K. (2019). Human-cooperative control of a wearable walking exoskeleton for enhancing climbing stair activities. IEEE Trans. Ind. Electr. 67, 3086–3095. 10.1109/TIE.2019.291457327295638

[B11] Li Z. Zhao K. Zhang L. Wu X. Zhang T. Li Q.. (2020). Human-in-the-loop control of a wearable lower limb exoskeleton for stable dynamic walking. IEEE/ASME Trans. Mechatron. 26, 2700–2711. 10.1109/TMECH.2020.304428927295638

[B12] Lu Z. Wang N. Yang C. (2021a). A constrained dmps framework for robot skills learning and generalization from human demonstrations. IEEE/ASME Trans. Mechatron. 26, 3265–3275. 10.1109/TMECH.2021.305702227295638

[B13] Lu Z. Wang N. Yang C. (2021b). A novel iterative identification based on the optimised topology for common state monitoring in wireless sensor networks. Int. J. Syst. Sci. 53, 1–15. 10.1080/00207721.2021.1936275

[B14] Malhan R. K. Joseph R. J. Shembekar A. V. Kabir A. M. Bhatt P. M. Gupta S. K. (2020). Online grasp plan refinement for reducing defects during robotic layup of composite prepreg sheets, in 2020 IEEE International Conference on Robotics and Automation (ICRA) (Paris: IEEE), 11500–11507.

[B15] Malhan R. K. Shembekar A. V. Kabir A. M. Bhatt P. M. Shah B. Zanio S.. (2021). Automated planning for robotic layup of composite prepreg. Robot. Comput. Integr. Manuf. 67:102020. 10.1016/j.rcim.2020.102020

[B16] Mandlekar A. Xu D. Martín-Martín R. Zhu Y. Fei-Fei L. Savarese S. (2020). Human-in-the-loop imitation learning using remote teleoperation. arXiv preprint arXiv:2012.06733.

[B17] Manschitz S. Gienger M. Kober J. Peters J. (2018). Mixture of attractors: A novel movement primitive representation for learning motor skills from demonstrations. IEEE Robot. Automat. Lett. 3, 926–933. 10.1109/LRA.2018.279253127295638

[B18] Manschitz S. Kober J. Gienger M. Peters J. (2014). Learning to sequence movement primitives from demonstrations, in 2014 IEEE/RSJ International Conference on Intelligent Robots and Systems (Chicago, IL: IEEE), 4414–4421.

[B19] Ochoa H. Cortesao R. (2021). Impedance control architecture for robotic-assisted mold polishing based on human demonstration. IEEE Trans. Ind. Electron. 69, 3822–3830. 10.1109/TIE.2021.307331027295638

[B20] Papadopoulos E. Aghili F. Ma O. Lampariello R. (2021). Robotic manipulation and capture in space: a survey. Front. Robot. AI 228:686723. 10.3389/frobt.2021.686723 34350212 PMC8326842

[B21] Pastor P. Hoffmann H. Asfour T. Schaal S. (2009). Learning and generalization of motor skills by learning from demonstration, in 2009 IEEE International Conference on Robotics and Automation (Kobe: IEEE), 763–768.

[B22] Peternel L. Oztop E. Babič J. (2016). A shared control method for online human-in-the-loop robot learning based on locally weighted regression, in 2016 IEEE/RSJ International Conference on Intelligent Robots and Systems (IROS) (Daejeon: IEEE), 3900–3906.

[B23] Peternel L. Petrič T. Babič J. (2015). Human-in-the-loop approach for teaching robot assembly tasks using impedance control interface, in 2015 IEEE International Conference on Robotics and Automation (ICRA) (Seattle, WA: IEEE), 1497–1502.

[B24] Raessa M. Chen J. C. Y. Wan W. Harada K. (2020). Human-in-the-loop robotic manipulation planning for collaborative assembly. IEEE Trans. Automat. Sci. Eng. 17, 1800–1813. 10.1109/TASE.2020.297891727295638

[B25] Ravichandar H. Polydoros A. S. Chernova S. Billard A. (2020). Recent advances in robot learning from demonstration. Ann. Rev. Control Robot. Auton. Syst. 3, 297–330. 10.1146/annurev-control-100819-06320632095878

[B26] Rigter M. Lacerda B. Hawes N. (2020). A framework for learning from demonstration with minimal human effort. IEEE Robot. Automat. Lett. 5, 2023–2030. 10.1109/LRA.2020.297061927295638

[B27] Rodrıguez I. Nottensteiner K. Leidner D. Kaßecker M. Stulp F. Albu-Schäffer A. (2019). Iteratively refined feasibility checks in robotic assembly sequence planning. IEEE Robot. Automat. Lett. 4, 1416–1423. 10.1109/LRA.2019.289584527295638

[B28] Santos L. Cortesão R. (2018). Computed-torque control for robotic-assisted tele-echography based on perceived stiffness estimation. IEEE Trans. Automat. Sci. Eng. 15, 1337–1354. 10.1109/TASE.2018.279090027295638

[B29] Saveriano M. Franzel F. Lee D. (2019). Merging position and orientation motion primitives, in 2019 International Conference on Robotics and Automation (ICRA) (Montreal, QC: IEEE), 7041–7047.

[B30] Si W. Wang N. Yang C. (2021a). Composite dynamic movement primitives based on neural networks for human-robot skill transfer. Neural Comput. Appl. 1–11. 10.1007/s00521-021-05747-8

[B31] Si W. Wang N. Yang C. (2021b). Reactive and constrained motion primitive merging and adaptation, in 2021 26th International Conference on Automation and Computing (ICAC) (Portsmouth: IEEE), 1–6.

[B32] Si W. Wang N. Yang C. (2021c). A review on manipulation skill acquisition through teleoperation-based learning from demonstration. Cognit. Comput. Syst. 3, 1–16. 10.1049/ccs2.12005

[B33] Tavakoli M. Carriere J. Torabi A. (2020). Robotics, smart wearable technologies, and autonomous intelligent systems for healthcare during the covid-19 pandemic: an analysis of the state of the art and future vision. Adv. Intell. Syst. 2, 2000071. 10.1002/aisy.20200007125855820

[B34] Wang N. Chen C. Yang C. (2020). A robot learning framework based on adaptive admittance control and generalizable motion modeling with neural network controller. Neurocomputing 390, 260–267. 10.1016/j.neucom.2019.04.100

[B35] Yang C. Chen C. He W. Cui R. Li Z. (2018). Robot learning system based on adaptive neural control and dynamic movement primitives. IEEE Trans. Neural Netw. Learn. Syst. 30, 777–787. 10.1109/TNNLS.2018.2852711 30047914

[B36] Yang C. Huang D. He W. Cheng L. (2021a). Neural control of robot manipulators with trajectory tracking constraints and input saturation. IEEE Trans. Neural Netw. Learn. Syst. 32:4231–4242. 10.1109/TNNLS.2020.3017202 32857705

[B37] Yang C. Peng G. Cheng L. Na J. Li Z. (2019). Force sensorless admittance control for teleoperation of uncertain robot manipulator using neural networks. IEEE Trans. Syst. Man Cybern. Syst. 51, 3282–3292. 10.1109/TSMC.2019.292087027295638

[B38] Yang C. Zeng C. Zhang J. (2021b). Robot Learning Human Skills and Intelligent Control Design. Boca Raton, FL: CRC Press.

[B39] Yang G.-Z. Nelson B. J. Murphy R. R. Choset H. Christensen H.. (2020). Combating covid-19–the role of robotics in managing public health and infectious diseases. Sci. Robot. 5, eabb5589. 10.1126/scirobotics.abb5589 33022599

[B40] Zeng C. Su H. Li Y. Guo J. Yang C. (2021). An approach for robotic leaning inspired by biomimetic adaptive control. IEEE Trans. Ind. Inform. 18, 1479–1488. 10.1109/TII.2021.308733727295638

[B41] Zeng C. Yang C. Chen Z. Dai S.-L. (2018). Robot learning human stiffness regulation for hybrid manufacture. Assembly Automat. 38, 539–547. 10.1108/AA-02-2018-019

[B42] Zeng C. Yang C. Cheng H. Li Y. Dai S.-L. (2020). Simultaneously encoding movement and semg-based stiffness for robotic skill learning. IEEE Trans. Ind. Inform. 17, 1244–1252. 10.1109/TII.2020.298448227295638

[B43] Zhang Y. Li M. Yang C. (2021). Robot learning system based on dynamic movement primitives and neural network. Neurocomputing 451, 205–214. 10.1016/j.neucom.2021.04.03430047914

[B44] Zhao T. Deng M. Li Z. Hu Y. (2018). Cooperative manipulation for a mobile dual-arm robot using sequences of dynamic movement primitives. IEEE Trans. Cogn. Dev. Syst. 12, 18–29. 10.1109/TCDS.2018.286892127295638

